# Glucose Controls Morphodynamics of LPS-Stimulated Macrophages

**DOI:** 10.1371/journal.pone.0096786

**Published:** 2014-05-05

**Authors:** Gerda Venter, Frank T. J. J. Oerlemans, Mietske Wijers, Marieke Willemse, Jack A. M. Fransen, Bé Wieringa

**Affiliations:** Department of Cell Biology, Nijmegen Centre for Molecular Life Sciences, Radboud University Medical Centre, Nijmegen, The Netherlands; University of Nebraska Medical Center, United States of America

## Abstract

Macrophages constantly undergo morphological changes when quiescently surveying the tissue milieu for signs of microbial infection or damage, or after activation when they are phagocytosing cellular debris or foreign material. These morphofunctional alterations require active actin cytoskeleton remodeling and metabolic adaptation. Here we analyzed RAW 264.7 and Maf-DKO macrophages as models to study whether there is a specific association between aspects of carbohydrate metabolism and actin-based processes in LPS-stimulated macrophages. We demonstrate that the capacity to undergo LPS-induced cell shape changes and to phagocytose complement-opsonized zymosan (COZ) particles does not depend on oxidative phosphorylation activity but is fueled by glycolysis. Different macrophage activities like spreading, formation of cell protrusions, as well as phagocytosis of COZ, were thereby strongly reliant on the presence of low levels of extracellular glucose. Since global ATP production was not affected by rewiring of glucose catabolism and inhibition of glycolysis by 2-deoxy-D-glucose and glucose deprivation had differential effects, our observations suggest a non-metabolic role for glucose in actin cytoskeletal remodeling in macrophages, e.g. via posttranslational modification of receptors or signaling molecules, or other effects on the machinery that drives actin cytoskeletal changes. Our findings impute a decisive role for the nutrient state of the tissue microenvironment in macrophage morphodynamics.

## Introduction

Macrophages are present in all tissues where they provide a first line of defense against pathogens and help to maintain steady-state tissue homeostasis by eliminating foreign matter and apoptotic cells via phagocytosis [1], [2]. To exert these functions they migrate and constantly survey their immediate environment for signs of tissue damage or presence of invading organisms [1]. During surveillance, danger signals are detected through Toll-like receptors (TLRs), intracellular pattern recognition receptors (PRRs) and interleukin(IL)-receptors [2]. When macrophages encounter stimuli like inflammatory cytokines (IFN-γ, TNF, or IL-4), foreign material (e.g. lipopolysaccharide; LPS), or immunoglobulin G (IgG) immune complexes, tissue-resident macrophages become activated to undergo a phenotypic change towards a classically activated M1 or alternatively activated (suppressive) M2 polarization state [1], [3], [4], which is accompanied by metabolic adaptation. Because M1 and M2 phenotypes represent extremes in a continuum of phenotypes that macrophages can adopt, we still have no clear picture of the (possibly reciprocal) relationship between their metabolic profile and activation state. The prevailing idea is that, in the resting state, macrophages utilize glucose at a high rate and convert 95% of it to lactate [5]. Upon polarization towards a M1 phenotype (e.g. after stimulation with LPS) glucose import via GLUT, as well as the glycolytic flux, is even further upregulated [5]–[7]. M2 macrophages, on the other hand, do not undergo such extensive metabolic change but have a metabolic profile comparable to that of unstimulated cells, with higher TCA-cycle and oxidative activity [5], [8]. Recently, Haschemi et al. [7] have shown that carbohydrate kinase-like protein (CARKL) orchestrates macrophage activation through metabolic control. CARKL overexpression drove cells towards an oxidative state and sensitized macrophages towards a M2 polarization state, while CARKL-loss promoted a rerouting of glucose from aerobic to anaerobic metabolism and induced a mild M1 phenotype. Conversely, Tannahill et al. [9] have demonstrated that LPS stimulation of macrophages causes an increase in the intracellular TCA-cycle intermediate succinate, which stabilizes M1-associated HIF-1α and thereby regulates the expression of the pro-inflammatory cytokine IL-1β.

Besides overall metabolic versatility, macrophages also exhibit a wide range of morphodynamic activities, needed to exert their tasks in tissue surveillance and host defense. To control these activities before and after polarization, macrophages continuously form actin-rich membrane protrusions and extend filopodia from their cell surface [10], [11]. Changes in the organization of the actin cytoskeleton thereby enable the cell to dynamically adapt its morphology to suit its particular function and differentiation state. For example, LPS induces polymerization of cytoskeletal actin filaments, cell spreading, and the formation of filopodia, lamellipodia, and membrane ruffles in monocytes and macrophages [12], [13]. Likewise, IL-4, which is released during tissue injury, causes the rearrangement of actin-rich podosomes to form rosettes in M2 macrophages, enabling degradation of-and migration through-dense extracellular matrices [14].

The rearrangements of cytoskeletal actin filaments that steer this behavior comprise multiple steps, including the nucleation and elongation of new filaments from ATP-bound G-actin monomers, the addition of these monomers to the barbed ends of existing filaments, the hydrolysis of actin-bound ATP within the growing filament, and the dissociation of ADP-G-actin at the pointed end [15]–[18]. ADP on liberated G-actin is substituted with ATP, producing new ATP-G-actin monomers for incorporation. The ensemble of activities in this complex process is regulated by more than a hundred actin-associated proteins (ABPs), several of which are influenced by the availability of ATP. Metabolism, specifically the binding and hydrolysis of ATP and ATP-ADP exchange, thereby not only dictates the behavior of the actin filaments themselves [19]–[21], but also controls the activity of regulatory factors like the Arp2/3 protein complex [22], [23], or upstream signaling such as the ROCK/Rho-GTPase pathway [24]. The idea that actin remodeling is a major cellular energy drain is corroborated by the observation that actin filaments are stabilized under conditions of global ATP-depletion in order to prevent ATP-hydrolysis within the filament and, thereby, ATP-consumption [25], [26].

Besides ATP availability [27], [28], intracellular pH and NAD(P)^+^/NAD(P)H ratio are other key metabolic parameters that influence actin network dynamics and cell motility [29], either by modifying actin itself [30]–[32] or by regulating the activity of proteins such as the actin depolymerizing factor cofilin, mical, and the actin severing protein gelsolin [33]–[35]. In most mammalian cells, production of ATP, NAD^+^/NADH, and H^+^ is dominated by carbohydrate catabolism via glycolysis and mitochondrial TCA cycle/oxidative phosphorylation (OXPHOS) pathways. Importantly, various glycolytic pathway enzymes that handle these metabolites/cofactors appear compartmentalized and are found associated with the actin cytoskeleton and with actin dependent cellular structures, such as pseudopodia, membrane ruffles, and lamellipodia [36]–[39]. Indeed, this coupling helps to explain the dependency of cell motility on glycolysis [40], [41]. Also phagocytosis by macrophages depends on glycolysis [42]–[44], but not much work has been done to study this association in detail. Seeing their vital role in host defense and maintenance of tissue homeostasis and their surprising versatility in adaptation of functioning in many different tissue environments, we wondered whether there is a link between the activation of glucose metabolism and the extent of morphodynamic change that macrophages undergo. To apprehend the coupling between actin cytoskeletal remodeling and metabolic state we here investigated the ability of LPS-stimulated (M1) RAW 264.7 and Maf-DKO macrophages to maintain functional activity under conditions where they were forced to shift between a (anaerobic) glycolytic or oxidative metabolism. RAW 264.7 and Maf-DKO cell lines were chosen because they are *in vitro* manipulable models that have retained marked plasticity to stimulus-directed polarized activation, but lack the phenotypic heterogeneity that is characteristic for primary macrophages [45], [46]. We report on a stringent dependency of morphodynamics of LPS-stimulated macrophages upon sufficient glucose supply.

## Materials and Methods

### Reagents

All reagents were obtained from Sigma-Aldrich (St. Louis, MO, USA), unless stated otherwise.

### Cell Culture

RAW 264.7 cells (kind gift from Dr. Hong-Hee Kim, Department of Cell and Developmental Biology, School of Dentistry, Seoul National University, Korea; [47]) were maintained in high-glucose DMEM (Gibco, Life Technologies, Paisley, UK) supplemented with 10% heat inactivated FBS (PAA laboratories, Pasching, Austria), 1 mM sodium pyruvate, and 4 mMGlutaMAX (Gibco, Life Technologies, Paisley, UK), at 37°C in a humidified atmosphere with 7.5% CO_2_. Maf-DKO cells (kind gift from Dr. Michael H. Sieweke, Centre d’Immunologie de Marseille-Luminy (CIML), Université Aix-Marseille, France; [46]) were maintained in the same way except that medium was supplemented with 20% conditioned medium from L929-cells containing macrophage colony stimulating factor (M-CSF).

### DNA Constructs and Transfection

Plasmid pEYFP-N1-ΔATG-Lifeact was constructed as follows: Lifeact [48] cDNA, containing human codon sequences flanked by a 5′ BglII and 3′ EcoRI restriction site, was commercially synthesized and ligated in a pUC57 plasmid by GenScript USA Inc. A forward primer (5′-CT CAG ATC TCC ACC *ATG* GGC GTG GCC GAC C-3′) was designed to introduce a BglII site and a Kozak sequence in front of the Lifeact start codon. Use of this primer together with the M13 universal reverse primer enabled amplification of the Lifeact encoding insert from pUC57 by PCR. PCR products were digested with BglII and EcoRI and ligated into pEYFP-N1-ΔATG plasmid DNA (pEYFP-N1 from Clontech with ATG on position 679 mutated to GCG).

For transfection, RAW 264.7 cells were seeded in 6 well plates at 300,000 cells/well and incubated overnight. Plasmid pEYFP-N1-ΔATG-Lifeact DNA (12 µg; linearized with *AseI*) was diluted in 1 ml serum-free DMEM and incubated for 20 minutes at 37°C with 24 µl Targefect-RAW transfection reagent (Targeting Systems, El Cajon, CA, USA). Transfection complexes (250 µl) were added to wells containing 2 ml fresh culture medium and incubated for 4 hours at 37°C after which medium was refreshed. A stable cell pool was established by culturing cells for two weeks in medium containing 500 µg/ml G418, followed by limited dilution cloning.

### Media for Metabolic Manipulation

For cell culture under glucose free conditions, glucose-free DMEM (Gibco, Life Technologies, Paisley, UK) was supplemented with 10 mM galactose. As control, medium supplemented with 25 mM glucose was used. Low glucose medium was prepared by supplementing glucose free DMEM with 1 mM glucose and 10 mM galactose (gluc/gal). Media were further supplemented with 10% heat inactivated dialyzed FBS, 1 mM sodium pyruvate, and 4 mM GlutaMAX. For inhibition of glycolysis, high (25 mM) glucose DMEM containing 10 mM 2-deoxy-D-glucose (2-DG), 10% heat inactivated FBS, 1 mM sodium pyruvate, and 4 mMGlutaMAX was used. Inhibition of OXPHOS was achieved by adding 2.5 µM oligomycin to normal culture medium (containing 25 mM glucose). For live imaging, phenol red-free media were prepared using DMEM Base powder from Sigma-Aldrich to which sodium bicarbonate, GlutaMAX, sodium pyruvate, glucose and/or galactose, 2-DG, or oligomycin were added.

### Proliferation Assay

For cell proliferation analysis, the protocol developed by Skehan et al. [49] was used. RAW 264.7 cells were seeded in four 96-well plates (30,000 cells/well) in 100 µl culture medium and incubated for eight hours. At T0, plates were washed once and the medium in plates T6, T15, and T24 was replaced with either control, galactose, gluc/gal, 2-DG, or oligomycin medium containing 100 ng/ml LPS, while plate T0 was fixed for sulforhodamine B (SRB) staining of protein content. After 0, 6, 15, and 24 hours, cells were washed twice with cold PBS and fixed with 10% trichloroacetic acid (TCA; J.T.Baker, Deventer, Holland) for 1 hour at 4°C. After fixation, plates were washed five times with water and stored at −20°C until all plates were collected. Cellular protein was stained with 50 µl 0.5% SRB in 1% acetic acid for 20 minutes after which wells were washed four times with 1% acetic acid. Plates were dried at 60°C for 3 hours, protein was dissolved in 150 µl 10 mM Tris-HCl (pH 10.5), and the absorbance of each well was measured at 510 nm on a BioRad Benchmark Plus micro plate reader. Values were corrected for background SRB staining by subtracting the average absorbance value of wells that contained medium only, from that of wells with cells.

### Apoptosis Assay

Apoptosis of RAW 264.7 cells was measured using a biosensor (pSIVA) developed by Kim et al. [50] (generous gift from Dr. Ralf Langen, University of Southern California). Briefly, cells (20,000/well) were seeded in a BD Falcon 96-well imaging plate and incubated overnight. On the day of assay, wells were washed once and medium was replaced with phenol red-free control, galactose, 2-DG, or oligomycin medium containing 8 ng/µl pSIVA or non-labeled control. The plate was immediately imaged for 24 hours, continuously, on a BD Pathway high-content spinning disc confocal microscope equipped with a temperature and CO_2_ controllable incubation chamber, using a 20x objective and 2×2 montage capture. Three wells were imaged per condition and the amount of apoptosis was determined by analyzing the increase in GFP-signal. For each well the threshold of the whole GFP-image series was adjusted and the total pixel area/frame was determined using Fiji imaging software [51] and plotted against time.

### ATP Assay

Intracellular ATP was determined using the CellTiter-Glo cell viability assay kit from Promega.

500,000 cells (RAW 264.7) were seeded per well of a 6-well plate and incubated in control medium, galactose medium or 1 mM glucose medium for 4 and 14 hours, 2-DG medium for 1 and 3 hours, or oligomycin-containing medium for 0.5 or 24 hours prior to assay. Cells were washed twice with ice cold PBS and then scraped in 350 µl ice cold 0.6 M perchloric acid (PCA). PCA extracts were centrifuged for 3 minutes at 4000 rpm and 4°C to pellet all cellular protein. Supernatants were neutralized with 140–155 µl 2 M KOH/0.2 M KH_2_PO_4_, pH 7.5 and diluted 1∶10 in water. Per well, 100 µl diluted PCA extract was added to 100 µl CellTiter-Glo reagent and the luminescence intensity per well was measured on a LUMIstar OPTIMA microplate luminometer. The ATP concentration was determined using an ATP standard series. For determination of total cellular protein, pellets were dissolved in 250 µl 1 M NaOH and heated for 30 minutes at 95°C. Protein concentration was then measured in 1∶50 diluted NaOH extracts.

### Glucose and Lactate Assays

Glucose consumption measurements were based on the Amplex Red Glucose/Glucose Oxidase assay kit from Molecular probes (Life Technologies, Eugene, Oregon, USA). Glucose, glucose oxidase, and Amplex Red reagent were used from the kit but horseradish peroxidase was obtained from Sigma-Aldrich and 1x reaction buffer was replaced with 0.05 M Tris-HCl, pH 7.5. Otherwise, the kit protocol was followed as described by the manufacturer. Lactate production was measured using the same protocol as for glucose consumption but replacing glucose oxidase with lactate oxidase and including a lactate standard series instead of glucose. RAW 264.7 cells were seeded in 12 well tissue culture plates and incubated for 6 or 24 hours in either 1 ml control or 1 ml 2.5 µM oligomycin medium. For glucose measurements, medium containing 5 mM glucose was used while lactate production was measured for cells grown in 25 mM glucose medium. For measurement of glucose consumption or lactate production during the last 6 hours of the 24 hour incubation period, medium was refreshed after 18 hours in one of the 24 hour plates. Prior to addition of incubation media, wells were always rinsed with pure DMEM containing no glucose. Medium was collected at the end of the 6 or 24 hour incubation period and supernatants were snap frozen in liquid nitrogen and stored at −20°C until analysis. Cytosolic extracts were prepared in lysis buffer (50 mM Tris-HCl pH 7.5, 100 mM NaCl, 5 mM MgCl_2_, and 0.5% NP-40; 4°C) and total protein was determined with the Bradford assay. Glucose consumption was calculated by subtracting the amount of glucose in the sample from that in medium without cells. Lactate production was calculated by subtracting the concentration of any lactate in the medium without cells from that of the samples. Glucose and lactate assays were performed in parallel.

### Oxygen Consumption Measurements

Mitochondrial respiration was assessed by measuring oxygen consumption on an Oroboros Oxygraph-2k respirometer according to a standard protocol provided by the manufacturer. RAW 264.7 and Maf-DKO cells were analyzed in parallel in two separate chambers of the respirometer. After air calibration of medium in the chambers and stabilization of the signal, 1×10^6^ cells (in 60 µl medium) were injected into the respective chambers. Basal respiration rate was measured at the point where the O_2_-flux signal stabilized. Oligomycin (2.5 µM) was added to each chamber and the leak respiration rate was determined after stabilization of the signal. Next, 7 µM carbonyl cyanide-*p*-trifluoromethoxyphenylhydrazone (FCCP, a mitochondrial uncoupler) was added to reach maximal oxygen consumption in the cells. Finally, 30 nM rotenone was added and after stabilization of the system, the residual oxygen consumption could be determined. The data were analyzed using the DatLab software provided with the instrument.

### Cellular Actin Staining

RAW 264.7 cells on coverslips were pre-incubated in control or 2-DG medium (3 hours), gluc/gal medium (4 and 24 hours), galactose medium (4 and 24 hours), or oligomycin-containing medium (0.5 and 24 hours) and stimulated with 100 ng/ml LPS overnight or left unstimulated. Medium was removed and cells were immediately fixed in 2% paraformaldehyde in 0.2 M sodium phosphate buffer for 30 minutes. Coverslips were washed twice with PBS and twice with PBS containing 20 mM glycine (MP Biomedicals, Illkirch Cedex, France; PBS-G) before permeabilization with 0.1% saponine/PBS-G for 20 minutes. This was followed by actin staining with Alexa 568-labeled phalloidin (1∶600 in 0.1% saponine/PBS-G) for 1 hour. Cells were successively washed four times for 2–4 minutes with 0.1% saponine/PBS-G and once with PBS alone. Coverslips were removed from wells, rinsed once in water, air dried, and imbedded in MoWiol on microscope slides. Z-scans consisting of 25×0.5 µm sections and the pinhole adjusted to one airy unit were recorded on a Zeiss LSM510 META confocal laser scanning microscope and merged to one single image using Fiji imaging software. Coverslips were prepared in duplo and per condition twelve different fields were analyzed.

### Scanning Electron Microscopy

Cells were seeded on 12 mm glass coverslips in 24-well plates and pre-incubated in control or galactose medium (4 hours), 2-DG medium (3 hours), oligomycin medium (0.5 and 24 hours), or gluc/gal medium (4 hours). In addition, cells were stimulated with 100 ng/ml LPS or left unstimulated. Cells were washed once with PBS and fixed with 2% glutarealdehyde in 0.1 M sodium cacodylate buffer for 1 hour. After washing cells twice with sodium cacodylate buffer, coverslips were stored in this buffer at 4°C until further fixation with 1% OsO_4_ (osmium tetroxide) for 30 minutes. Coverslips were then washed once with water and dehydrated in a graded series of alcohol washes. Finally, coverslips were critical point dried and mounted for scanning electron microscopy on a JEOL SEM6340F Field Emission Scanning Electron microscope. Coverslips were prepared in duplo and per condition 8–10 different fields were analyzed.

### Quantification of Filopodia and Cell Circumference

For quantification of filopodia, fluorescence microscopy or SEM images of fixed cells were analyzed. Lines were drawn to delineate contour segments of cells in areas where no contacts with neighboring cells were seen. The length and number of filopodia extending from each contour segment were determined. For phalloidin stained cells, 30–40 different line segments with a total contour length corresponding to the circumference of 20–50 cells, were included per condition. From the SEM images 20–26 line segments, with a total contour length corresponding to the circumference of 10–17 cells, were analyzed per condition.

To compare circumferences of whole cells, contour lengths of circular lines around individual cells were measured. Per condition, 20–50 cells were analyzed from both the fluorescence and electron microscopy image collection.

### Spreading Assay

RAW 264.7 cells expressing Lifeact-EYFP were stimulated with 100 ng/ml LPS overnight and simultaneously pre-incubated in control or galactose, 2-DG, oligomycin, or gluc/gal medium for 0, 3, 15, or 24 hours before they were harvested by treatment with 1 mM EDTA/PBS (10 min at 37°C). After washing, the cells were suspended in medium with 1% bovine serum albumin. A recovery period of 20 minutes at 37°C was allowed before cells were seeded in a fibronectin coated (50 µg/ml for 2 h at 37°C) BD Falcon 96-well imaging plate at 4,000 cells/well in the presence of 100 ng/ml LPS. Cell spreading was monitored for three hours by recording the increase in EYFP-pixel area per cell (20 cells/well) on a BD Pathway high-content spinning disc confocal microscope, using a 20x objective and 3×3 montage capture. In order to combine the data from three independent experiments (performed in duplicate), the time axes had to be synchronized. To achieve this, a Boltzmann simulation curve was produced for each data set between time points 0 and 200 in steps of 2 minutes using OriginLab data analysis and graphing software (OriginPro 6.1). Data sets were then combined and analyzed for statistical significance by applying a repeated measures analysis using PASW statistics 18 SPSS software. Three time frames (consisting of 30 data points each) of each curve were compared with the corresponding three time frames of the control curve.

### Phagocytosis Assay

Phagocytic activity was determined as zymosan ingestion capacity essentially as described by Kuiper et al. [27]. Zymosan particles were dissolved in PBS at 10 mg/ml and left to rehydrate for at least one hour. Next, zymosan was sonicated three times for 5 seconds, spun down, resuspended in sodium carbonate buffer (pH 9.6), sonicated, and incubated with 1 µg/ml fluorescein isothiocyanate (FITC) for 1 hour at room temperature, in the dark. After FITC labeling, zymosan was washed three times with sodium carbonate buffer and incubated in 1 M Tris HCl, pH 8.0, for 30 minutes. Zymosan was then washed twice with PBS and finally resuspended in PBS. After one more sonication step, FITC-labeled zymosan was divided in aliquots, frozen in liquid nitrogen, and stored at −20°C.

Phagocytosis assays were performed in 12-well plates in which cells were seeded the day before. The number of cells seeded was adjusted for some conditions, due to the inhibitory effect on proliferation. To compensate for reduced proliferation rate, 150,000 and 200,000 RAW 264.7 cells were seeded per well for 14 h and 24 h galactose treatment, respectively. For all other conditions 100,000 RAW 264.7 cells were seeded per well. For assays with Maf-DKO cells, 200,000 cells were seeded per well. Prior to assay, cells were pre-incubated in control or galactose medium (0, 4, and 14 hours), 2-DG medium (0, 1, and 3 hours), oligomycin medium (0, 0.5, 3, 15, and 24 hours), or gluc/gal medium (0, 4, and 14 hours), and activated overnight with 100 ng/ml LPS. For assays with Maf-DKO cells in galactose medium, L929-cell conditioned medium was omitted to ensure that no glucose was present in the medium. FITC-labeled zymosan particles were opsonized by incubation in fetal bovine serum for 1 hour at 37°C, washed twice with PBS, and finally resuspended in serum-free control, galactose-, 2-DG-, oligomycin-, or gluc/gal-medium. Cells were washed once with glucose-free DMEM and incubated with 1 ml zymosan suspension for 30 minutes at 37°C. The particle-to-cell ratio was approximately 10∶1. Particle engulfment was terminated after washing cells twice with PBS and removing extracellular zymosan by treatment with 500 µl 100 U/ml lyticase for 10 minutes at room temperature. Successively, cells were detached with 0.05% trypsin/0.5 mM EDTA (Gibco, Life Technologies, Paisley, UK), resuspended in 1 ml medium with serum, pelleted, and finally resuspended in 200 µl 1% paraformaldehyde in PBS. Samples were analyzed by FACS (BD FACS Calibur) and phagocytosis activity was determined by measuring the percentage of FITC positive cells and the fluorescence intensity in these cells. The phagocytic index of each sample was then calculated as the product of the mean FITC intensity of the positive population times the % of FITC positive cells.

For the glucose rescue assay, 100,000 Maf-DKO and 60,000 RAW 264.7 cells were seeded per well in 24 well plates in medium containing 20% L929 cell conditioned medium and stimulated with 100 ng/ml LPS overnight. Cells were washed with glucose-free DMEM and incubated with FITC-COZ in galactose medium for 30 minutes at 37°C. Then, 1 mM glucose or glucose-free DMEM was added and the cells were incubated for another 30 minutes. Thereafter, cells were detached and prepared for FACS.

### Adhesion and Internalization Assay

Internalization efficiency was determined essentially as described by Sahlin et al (1983). RAW 264.7 cells and zymosan particles were prepared as for the phagocytosis assay. Pre-incubation in different media without or with inhibitors was as follows: galactose 0 hours; 2-DG, 1 hour; oligomycin, 0 and 24 hours; and gluc/gal, 0 hours. After 30 minute incubation with serum opsonized zymosan at 37°C, plates were transferred to ice and wells were washed twice with ice cold PBS. Cells were then scraped in 1 ml cold medium, divided in two equal portions, transferred to microcentrifuge tubes, and spun down. Cells in one portion were resuspended in 0.05% trypan blue in potassium dihydrogen citrate/saline, pH 4.4 to quench all extracellular FITC-COZ, and cells in the other portion were taken up in the same buffer but without trypan blue. Samples were analyzed by FACS.

### Statistical Analysis

Data were analyzed either with the Student’s t-test, one-sample t-test for relative values, or using a two-way ANOVA and the Bonferroni post-test (GraphPad software, Inc., Version 4). All values are expressed as mean+/−SEM. Values were considered to be significantly different when *p* values were <0.05.

## Results

### The Effect of Glycolysis and OXPHOS Inhibition on RAW 264.7 Cell Proliferation and Viability

**Figure 1:**
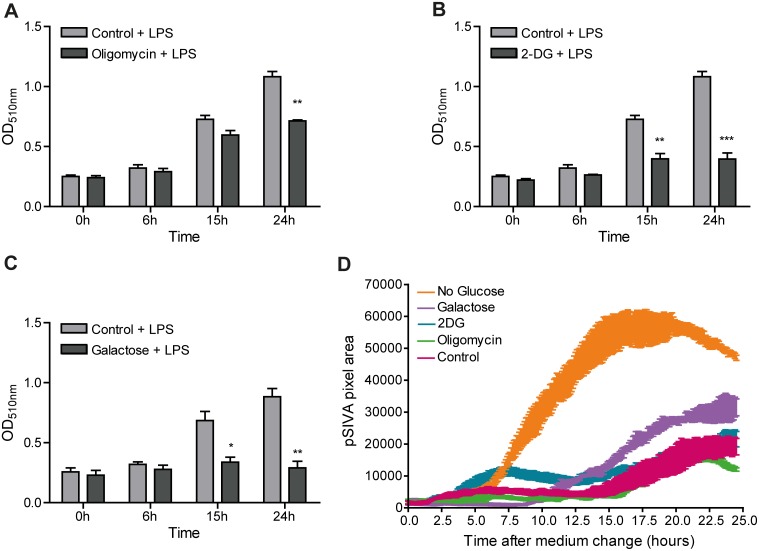
Proliferation and viability of RAW 264.7 cells under different metabolic conditions. Proliferation was monitored for 24 hours in the presence of LPS and expressed as the increase in total cellular protein in either control medium (25 mM glucose) or medium containing 2.5 µM oligomycin and 25 mM glucose (A), 10 mM 2-DG and 25 mM glucose (B), or 10 mM galactose and no glucose (C). Cell viability was also assessed for 24 hours under the same medium conditions in the presence of pSIVA apoptosis biosensor [50] (D). The appearance of the fluorescent pSIVA signal was recorded in real time and the total pixel area per frame was measured using Fiji Imaging software. An increase in the pSIVA pixel area correlates linearly with increase in the amount of apoptotic cells. Data in A–C represent means ± SEM of three independent experiments performed in triplicate and in D the averages of one experiment performed in triplicate. (*p<0.05, **p<0.01, ***p<0.001, unpaired t-test).

Tissue-resident macrophages, dependent on the niche that is occupied within the body, may become exposed to dramatically different nutrient conditions, including variation in oxygen and carbohydrate supply. Their functional plasticity relies thereby largely on the capacity to adapt and switch between carbohydrate metabolism via glycolysis or mitochondrial TCA cycle and OXPHOS reactions. For study of different aspects of this coupling we chose to focus on the well-established macrophage model, RAW 264.7 [45], and used another macrophage model, Maf-DKO cells [46], for comparison of the key findings. In order to find a range of conditions wherein the metabolic state of RAW 264.7 macrophages can be manipulated without compromising cell proliferation and viability, we monitored cells for a period of at least 24 hours in the presence of the complex V OXPHOS inhibitor oligomycin, the competitive glycolysis inhibitor 2-deoxy-D-glucose (2-DG), or in the absence of glucose. Cell proliferation was determined as the increase in total protein mass both in the absence and presence of LPS. Since the effect of metabolic inhibitors on cell proliferation appeared independent of this stimulation, we chose to present only results obtained in the presence of LPS (Figure 1A–C). Cell viability was monitored using the the pSIVA apoptosis biosensor with switchable fluorescence states (Figure 1D). When pSIVA binds to annexin V that is exposed on the surface of early apoptotic cells its fluorescence can be detected at an emission wavelength between 500 and 550 nm. In the absence of apoptotic cells, pSIVA remains unbound and is non-fluorescent and non-detectable, therefore, an increase in the pSIVA fluorescence signal (plotted as pSIVA pixel area on the y-axis in Figure 1D) correlates with an increase in the amount of apoptotic cells.

Incubation with 2.5 µM oligomycin caused a 60–70% inhibition of OXPHOS activity (measured as the decrease in O_2_-consumption, Figure S1) and a marked reduction in proliferation rate (Figure 1A) without inducing excessive apoptosis (Figure 1D). Inhibition of glycolysis with 10 mM 2-DG induced apoptosis at an early time point (i.e. after 3–4 hours) in a relatively small % of cells in the population and, under this condition, proliferation ceased (Figure 1B and 1D). Upon substitution of glucose with 10 mM galactose (glucose deprivation) apoptotic cells began to appear after about 10 hours (Figure 1D), in a pool of cells with already significantly reduced proliferation capacity (Figure 1C). These results imply that the proliferation of RAW 264.7 cells is dependent on both glycolysis and mitochondrial activity, but that viability is better preserved by glycolysis alone than by mitochondrial TCA/OXPHOS activity alone.

### ATP Metabolism during Metabolic Inhibition of RAW 264.7 Cells

**Figure 2:**
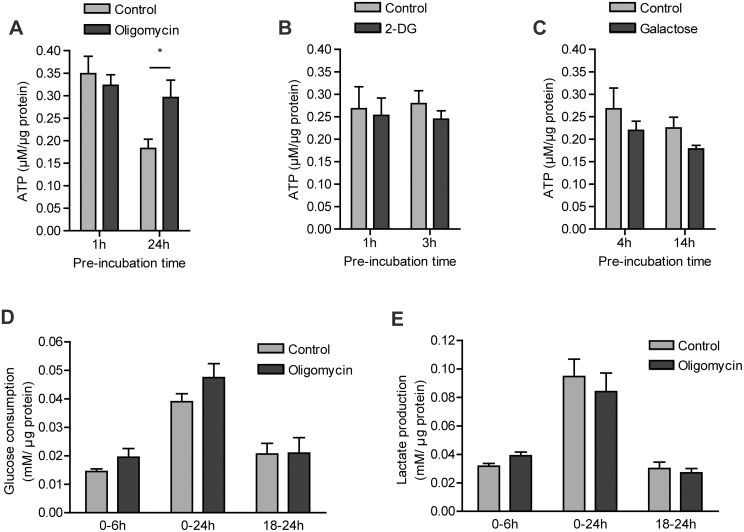
ATP levels under different metabolic conditions and the effect of OXPHOS inhibition on glycolytic flux. RAW 264.7 cells were incubated for the indicated time intervals with control (25 mM glucose) medium, or medium containing 2.5 µM oligomycin and 25 mM glucose (A,D,E), 10 mM 2-DG and 25 mM glucose (B), or 10 mM galactose and no glucose (C). Intracellular ATP concentrations and total cellular protein were measured in PCA cell extracts (A,B,C). Glucose consumption (D) and lactate production (E) were measured in medium supernatants during the first 6 hours (0–6 h), the last 6 hours (18–24 h), as well as the whole 24 hours (0–24 h) of treatment. Data represent means ± SEM of three experiments performed in triplicate. (*p<0.05, unpaired t-test).

Since macrophages produce cellular ATP mainly via glycolysis [52], [53], inhibition of this pathway or limitation of its starting substrate glucose may have a significant impact on cellular energy homeostasis if there is no adequate compensation by mitochondrial ATP production. To study whether macrophages are versatile enough to accommodate changes in ATP supply pathways, we first followed [ATP] in RAW 264.7 cells during early stages and modes of inhibition, under conditions where cells were still fully viable (1 hour oligomycin and 2-DG, 4 hours galactose). Comparison was drawn to [ATP] found at later stages of inhibition when either apoptosis was initiated (3 hours 2-DG and 14 hours galactose) or proliferation was significantly reduced (24 hours oligomycin). Compared to control cells, short term oligomycin treatment (3 h) did not affect ATP level. Upon prolonged incubation in presence of oligomycin (24 h) we even did not observe the 50% drop in ATP level that occurred in RAW 264.7 cells, presumably as a result of medium depletion. We currently explain this observation by assuming that continuous presence of oligomycin forces rewiring of (mitochondrial) metabolism and that this protects against this depletion. More study is needed to obtain the evidence to support this explanation. In the presence of 2-DG, cells managed to maintain ATP levels beyond the 3 hours before apoptosis was induced (Figure 2B). Also in the presence of galactose, i.e. under conditions of glucose deprivation, no marked decrease in cellular ATP was observed until 14 hours, compared to control cells (Figure 2C).

Under conditions where carbohydrate catabolism had to be rewired, i.e. under oligomycin treatment, we observed a trend towards higher glucose consumption by treated compared to control cells, although the difference was not statistically significant (Figure 2D). Oligomycin treatment did not, however, increase the flux of glucose to lactate (Figure 2E). Normally, already about 95% of all glucose consumed is converted into lactate and only a very small fraction of pyruvate is imported into the mitochondria in macrophages [5]. This explains why inhibition of OXPHOS cannot cause a significant increase in the turnover of pyruvate to lactate and why no significant increase in lactate production was observed.

### Deviant Actin Cytoskeleton and Cell Surface Morphology of M1 Macrophages in the Absence of Glucose

**Figure 3:**
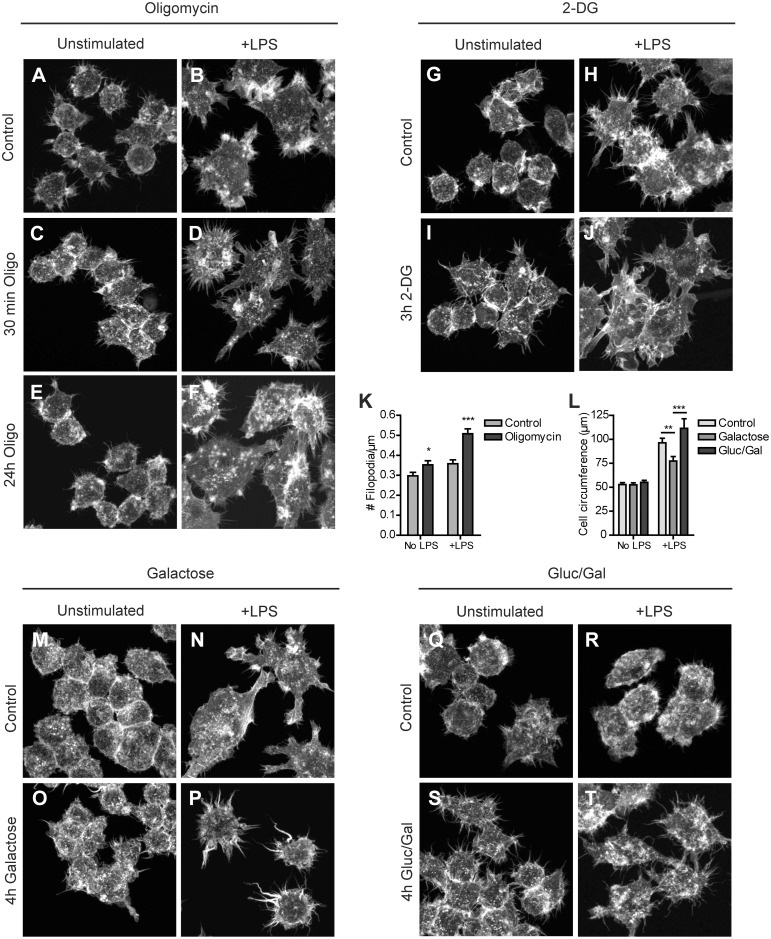
Actin cytoskeletal structural changes induced by inhibition of glycolysis or mitochondrial OXPHOS. RAW 264.7 cells were seeded on glass coverslips, incubated in control medium or medium containing 2.5 µM oligomycin and 25 mM glucose (A–F), 10 mM 2-DG and 25 mM glucose (G–J), 10 mM galactose and no glucose (M–P), or 1 mM glucose and 10 mM galactose (Q–T) for the indicated time periods and stimulated overnight with LPS or left unstimulated. After fixation in 2% PFA, cellular actin was stained with phalloidin-Alexa568 and cells were imaged on a Zeiss LSM510 meta confocal laser scanning microscope. The number of filopodia extending radially from the cell surface (expressed as # filopodia per µM contour length; see M&M) was determined for control cells and cells treated for 24 hours with oligomycin, in the presence and absence of LPS (K). The average cell circumference was determined for cells in control medium or medium containing 10 mM galactose, or 1 mM glucose and 10 mM galactose (L). (*p<0.05, **p<0.01, ***p<0.001, unpaired t-test).

**Figure 4:**
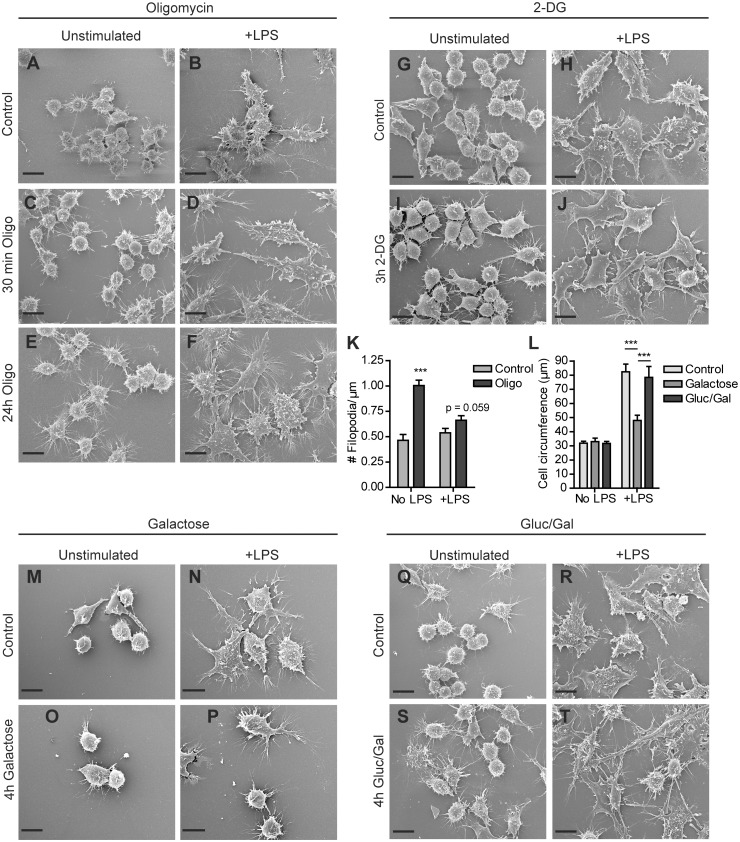
Effect of glucose deprivation and glycolysis or OXPHOS inhibition on morphology of RAW 264.7 cells. Cells were seeded on glass coverslips, incubated in control medium or medium containing 2.5 µM oligomycin and 25 mM glucose (A–F), 10 mM 2-DG and 25 mM glucose (G–J), 10 mM galactose and no glucose (M–P), or 1 mM glucose and 10 mM galactosel (Q–T) for the indicated time periods and stimulated overnight with LPS or left unstimulated. Coverslips were fixed and subjected to scanning electron microscopy. The number of filopodia extending radially from the cell surface was determined for control cells and cells treated for 24 hours with oligomycin, in the presence and absence of LPS (K). The average cell circumference was determined for cells in control medium or medium containing 10 mM galactose, or 1 mM glucose and 10 mM galactose (L). (***p<0.001, unpaired t-test). (Bar = 10 µm).

Polarization of macrophages towards a M1 phenotype with LPS is accompanied by an increase in glucose uptake and an accelerated conversion to lactate, while the rate of OXPHOS is reduced [5], [6]. Concomitantly, LPS induces extensive remodeling of the actin cytoskeleton of macrophages. *In vivo*, the collective changes in cell morphology and the formation of specific membrane structures, like filopodia and membrane ruffles on the surface of macrophages [12], [13] form the morphodynamic adjustments that help macrophages in adhering to the tissue matrix and in probing and capturing phagocytic targets. In order to determine whether the metabolic changes in LPS-stimulated macrophages have any true morphofunctional significance, we metabolically challenged RAW 264.7 cells, using conditions under which cells remained fully viable. First, we assessed the surface morphology and appearance of actin-based membrane protrusions, by scanning electron microscopy (SEM) and fluorescence microscopy of (phalloidin-stained) RAW 264.7 cells before and after LPS-stimulation. Oligomycin treated cells preserved the ability to spread upon LPS-stimulation and appeared to have more filopodia than control cells (Figure 3A–F and Figure 4A–F). These thorn-like protrusions became clearly visible after 6 hours (not shown) and were observed on both LPS stimulated and unstimulated cells. Quantification of the number of filopodia extending radially from the cell body after 24 hours, confirmed that oligomycin induced filopodia formation in RAW 264.7 cells (Figure 3K and 4K) although the extent of oligomycin effects (on cells with and without LPS) as determined by fluorescence and SEM microscopy varied. Partly this variation may be due to differential effects of the fixation-staining, and also to the semi-quantitative nature of protrusion recognition and scoring in the image analyses, for the light- and electron microscopy assays. Importantly, removing glucose from the culture medium markedly affected cell morphology. Although the actin cytoskeletal morphology of unstimulated galactose treated cells did not deviate much from control cells after 4 hours, LPS stimulated galactose treated cells had markedly different actin cytoskeletal morphology (Figure 3L–P). Instead of expanding their surface area and forming distinct protrusions, galactose cells stayed rounded and had a smaller cell circumference than control cells (Figure 3L; see also SEM results Figure 4L–P). When galactose-containing medium was supplemented with 1 mM glucose, the LPS-induced modifications of the cytoskeleton and surface morphology were similar to those seen in control cells (Figure 3L&Q–T and Figure 4L&Q–T). In contrast, inhibition of glycolysis with 2-DG did not alter the actin cytoskeleton or the cell morphology in either LPS- or unstimulated cells (Figure 3G–J and Figure 4G–J). In combination, our observations suggest that the presence or uptake of glucose, but not necessarily its use in metabolic breakdown, is involved in LPS-induced morphodynamic transitions in RAW 264.7 macrophages.

### LPS-induced Macrophage Spreading Depends on Constant Glucose Supply

**Figure 5:**
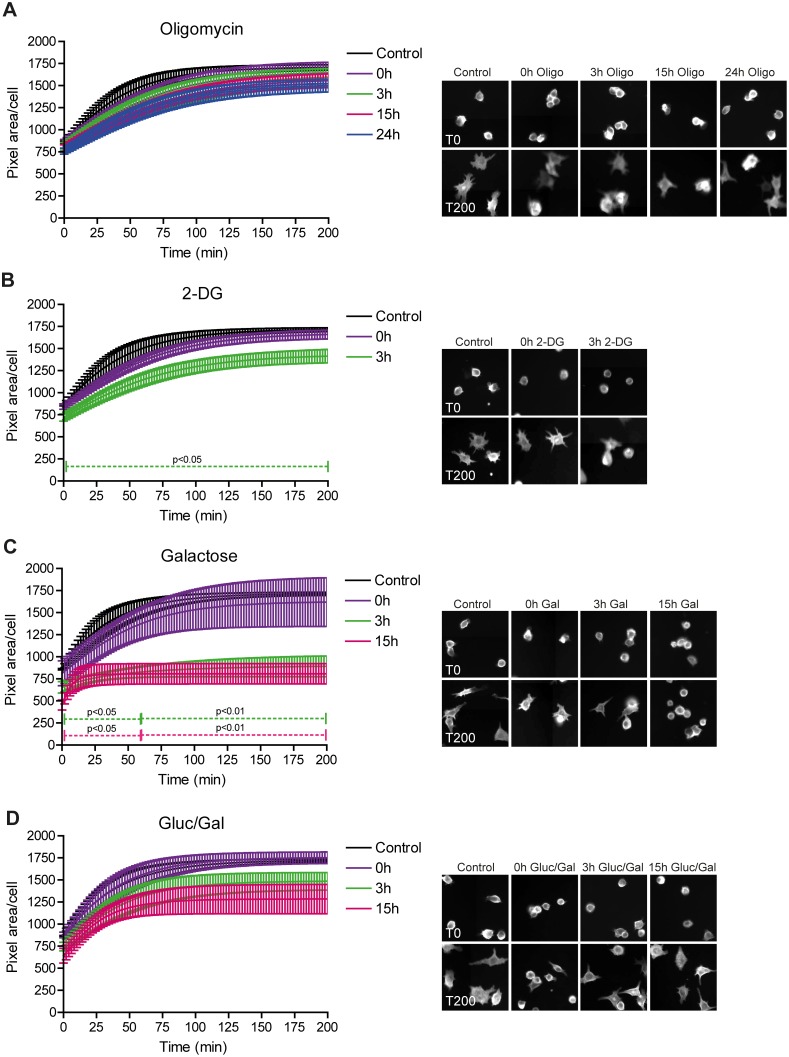
LPS-stimulated spreading of RAW 264.7 macrophages is compromised by glucose deprivation. RAW 264.7 macrophages expressing Lifeact-EYFP were pre-incubated in control medium or medium containing 2.5 µM oligomycin and 25 mM glucose (A), 10 mM 2-DG and 25 mM glucose (B), 10 mM galactose and no glucose (C), or 1 mM glucose and 10 mM galactosel (D) medium for the indicated time periods. To assess spreading efficiency, cells were detached with EDTA, re-suspended, seeded in 96 well plates and allowed to adhere. Cell spreading of EYFP-positive cells was recorded over time using a BD Pathway high content microscope. The average pixel area per cell was determined at 10 minute intervals. Lines and bars represent means ± SEM of three independent experiments performed in triplicate. For every condition, representative images of cells at 0 and 200 minutes are presented in the panel on the right.

Next, to further investigate the role of glucose in LPS-induced protrusive actin dynamics and obtain a more quantitative measure of the effect of the different metabolic conditions on global macrophage morphodynamics, we analyzed the spreading ability of RAW 264.7 cells on fibronectin-coated glass. During cell spreading, the increase in cell size is determined by global reorganization of the cellular actin cytoskeleton [54]. Inhibition of OXPHOS with oligomycin had no significant effect (Figure 5A), whereas 2-DG treatment caused slight retardation of spreading (Figure 5B). In contrast, removal of glucose from the medium and replacement with galactose dramatically inhibited surface expansion. Even without pre-incubation (0 h), cells spread less efficient in the absence of glucose. With pre-incubation in glucose-free medium, spreading was further inhibited and already significantly impaired after 3 hours of pre-treatment (Figure 5C). Typically, in the presence of 1 mM glucose cells largely retained the ability to expand their surface area (Figure 5D). Altogether, our findings are in keeping with the morphological observations and indicate that the availability of glucose is indispensable for LPS-induced spreading of macrophages.

### Phagocytosis of Complement-opsonized Zymosan by LPS-stimulated Macrophages is Fueled by Glycolysis and Requires the Presence of Extracellular Glucose

**Figure 6:**
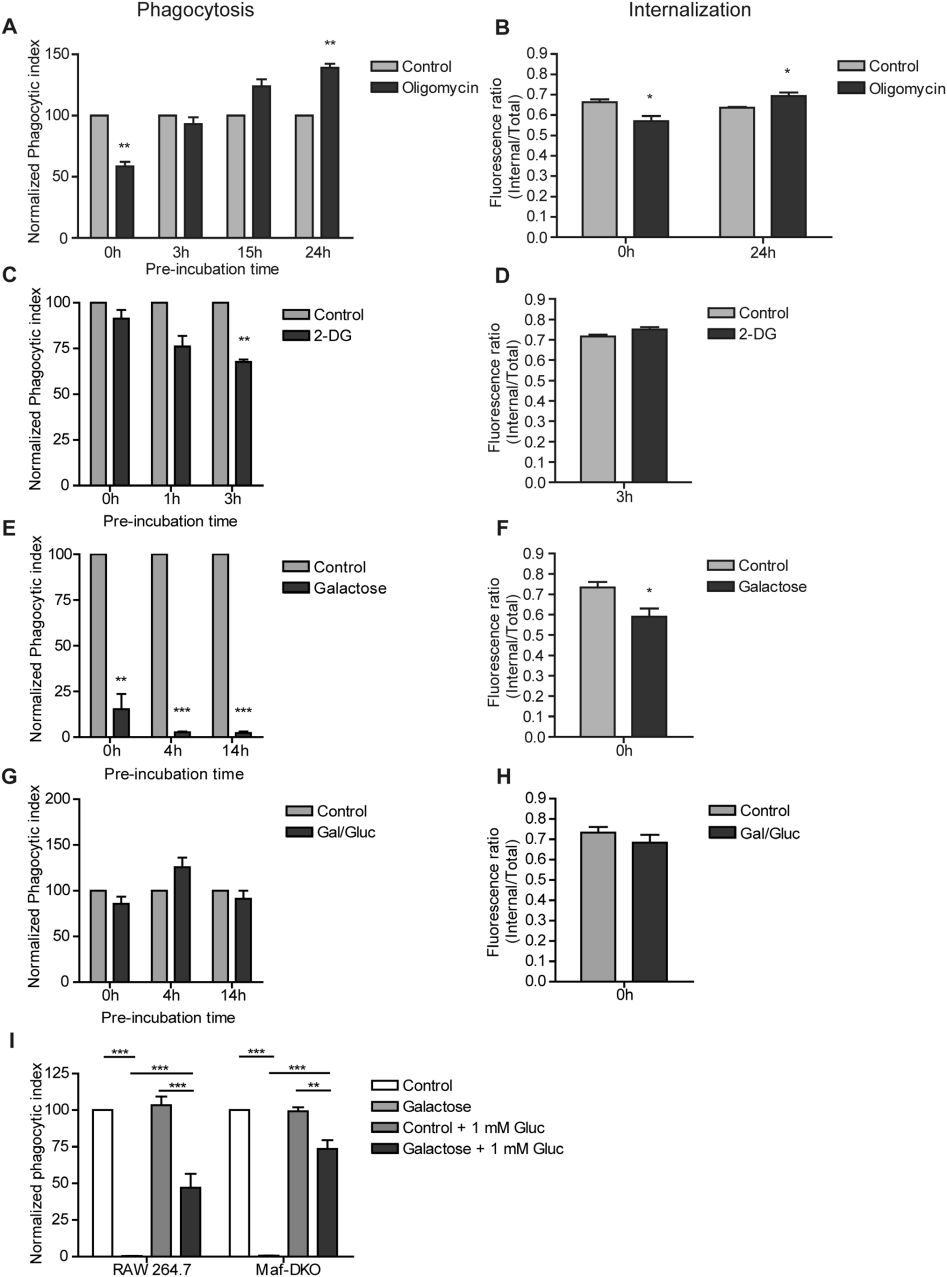
Macrophages require glucose for phagocytosis of COZ. RAW 264.7 cells were incubated for the indicated times with control medium, or medium containing 2.5 µM oligomycin and 25 mM glucose (A&B), 10 mM 2-DG and 25 mM glucose (C&D), 10 mM galactose and no glucose (E&F), or 10 mM galactose and 1 mM glucose (G&H) and stimulated o/n with 100 ng/ml LPS. The phagocytic index (A,C,E&G) was determined by incubating cells in the respective media with FITC-labeled complement opsonized zymosan (COZ) particles for 30 min, analyzed by FACS and calculated as described in materials and methods. The internalization efficiency (B,D,F&H) was determined by quenching extracellular FITC-COZ of one sample fraction with 0.05% trypan blue in potassium dihydrogen citrate/saline, pH 4.4. Unquenched fractions were used to determine the total fluorescence per cell (internalized and external particles), while quenched fractions were used to measure only the internal fluorescence per cell. The effect of reintroducing glucose (+1 mM Gluc) in the medium after 30 minutes of phagocytosis in glucose-free medium was assessed in both RAW 264.7 and Maf-DKO cells (I). Values in A,C,E,G&I represent normalized means ± SEM of three or four independent experiments performed in triplicate (*p<0.05, **p<0.01, ***p<0.001; one-sample t-test (A–H) or two-way ANOVA and Bonferroni posttest (I)). Values in B,D,F&H represent means ± SEM of three experiments performed in triplicate. (*p<0.05; unpaired t-test).

We finally examined whether metabolic conditions (with differential nutrient supply or inhibitor conditions as above) also affect phagocytosis, the main actin-dependent function of macrophages [27]. Formation of the phagocytic cup is driven by local polymerization of actin filaments, associated with membrane alterations and regionally confined topological alterations at the cell surface. Unlike cell spreading, particle uptake via phagocytosis, therefore, is localized and determined by mechanistic events that occur within the small area of contact between cell and particle. Acute inhibition (0 h pre-incubation) of OXPHOS initially led to a reduction in internalization efficiency as well as the overall phagocytic index of RAW 264.7 cells (Figure 6A, B), however, after three hours, phagocytosis efficiency was restored and with longer oligomycin treatment (24 h) phagocytic index and the particle internalization capacity was even higher than in control cells. In the presence of ample glucose (25 mM), competitive inhibition of glycolysis by 2-DG only inhibited phagocytosis significantly after pre-incubation (Figure 6C). 2-DG did not have an acute effect, but after 1 hour of 2-DG pre-incubation phagocytosis was markedly down (*p* = 0.055). This effect became even more pronounced with prolonged (3 h) pre-treatment, a period wherein cell viability was still not affected (Figure 1D). The efficiency of particle internalization was, however, not compromised (Figure 6D) by addition of 2-DG. Phagocytosis in the presence of oligomycin and 2-DG was also assessed in a second macrophage cell line (Maf-DKO cells). In these cells, oligomycin treatment had no effect at all, while 2-DG inhibited Maf-DKO phagocytosis to the same extend as in RAW 264.7 cells (Figure S2A, B).

Upon total glucose deprivation (by substitution with galactose), the phagocytic index in both RAW 264.7 and Maf-DKO cells dropped immediately below 20% (Figure 6E and Figure S2C). An even bigger drop was seen in RAW 264.7 cells after 4 and 14 hours (Figure 6E). It is important to note that at 0 and 4 hours the cells were still fully viable. Thus, we conclude that the reduction in phagocytic activity must be solely the result of insufficiency in glucose availability and not be a secondary effect of functional incapacitation. The low phagocytic index in the absence of glucose was accompanied by a reduction in particle internalization efficiency (Figure 6F) as well as a reduction in the binding capacity of the cells since the percentage of FITC-COZ positive cells was also reduced by more than 50% (results not shown). These results suggest that glucose availability is essential for both particle binding and internalization. Interestingly, and in keeping with this conclusion, supplementation of galactose medium with 1 mM glucose enabled the cells to maintain full phagocytosis capacity (Figure 6G&H).

Finally, we wanted to determine whether the inhibitory effect of acute glucose deprivation on phagocytosis could be directly rescued by reintroducing glucose. RAW 264.7 and Maf-DKO cells were incubated with FITC-COZ in galactose medium for 30 minutes after which 1 mM of glucose was added and cells were incubated for another 30 minutes. RAW 264.7 phagocytosis capacity was restored to 45% while Maf-DKO cells regained 75% of their phagocytosis capacity (Figure 6I). Taken together, our findings imply that glycolysis is critical for phagocytosis of COZ while OXPHOS activity is dispensable. Moreover, our results suggest that macrophages require the presence of glucose in order to successfully bind and internalize COZ particles.

## Discussion

Increased morphodynamic activity, facilitated by rearrangement of the actin cytoskeleton, is a cellular response characteristic to LPS-stimulated macrophages. This dynamic actin remodeling is essential to macrophage function and considered to be an energy draining process. In endothelial cells, actin-ATP hydrolysis accounts for almost a fifth (18%) of total ATP consumption [55] and in platelets and neurons it is estimated to be more than 50% [26], [56]. Cellular energy metabolism and actin-based cell dynamics are, therefore, tightly coupled processes. Against this backdrop LPS also induces a shift in macrophage redox metabolism, accompanied by increased fluxes through glycolysis as well the pentose phosphate pathway for NADPH production [5], [7]. Here we asked the question whether these temporal associations also involve a functional regulatory coupling between metabolic and morphodynamic changes in LPS stimulated macrophages.

Our results show that the contribution of mitochondrial OXPHOS to cellular energy production is significantly smaller than that of glycolysis and that mitochondrial OXPHOS activity is not essential for fueling of morphodynamic processes in macrophages. We found that oxygen consumption in resting RAW 264.7 and Maf-DKO cells is two to four times lower than, for example, in mouse embryonic fibroblasts (Figure S1; [57]). More direct support for the dispensability of mitochondrial metabolism comes from the observation that, even under conditions where we enforced an almost complete dependency on glycolysis by treatment with oligomycin, the ability of RAW 264.7 macrophages to undergo LPS-induced actin cytoskeleton remodeling remained intact. Although phagocytosis capacity in RAW 264.7 cells was initially inhibited upon acute oligomycin treatment, this was restored within three hours and did not occur in Maf-DKO cells at all. Our observations are consistent with an earlier study by Kvarstein [58], wherein it was shown that OXPHOS inhibition with oligomycin and antimycin only had an effect on phagocytosis when used in combination with 2-DG. Another study by Cifarelli et al. [43] showed minor inhibition of phagocytosis with sodium azide and 2–4-dinitrophenol only after 3 hours. Our findings additionally showed that OXPHOS has also a negligible role in other aspects of macrophage morphodynamics.

In contrast, glucose metabolism through glycolysis (the dominating metabolic route of LPS-stimulated macrophages) was clearly indispensable. Perturbation of this pathway was achieved either by using the glycolytic inhibitor 2-DG, or by replacing glucose with galactose in the culture medium, thereby forcing the cells to synthesize and use mitochondrial ATP instead of ATP obtained from glycolysis [59], [60]. Although 2-DG treatment did not affect the LPS-induced morphology of RAW 264.7 cells, it caused a minor inhibition of the spreading ability of these cells. Additionally, inhibition of glycolysis with 2-DG also led to a significant reduction in phagocytosis capacity. This has been reported before for leukocytes in general and peritoneal macrophages [42], [58], [61]. Here, we extend these observations and additionally show that phagocytosis of complement opsonized zymosan by both RAW 264.7 and Maf-DKO cells depends on the presence of glucose in the cultivation medium during the time of uptake. We know only of two other papers that have reported on direct effects of glucose deprivation. In these studies, phagocytosis of unopsonized *P.aeruginosa* by human and murine peritoneal and alveolar macrophages was shown to depend on the presence of glucose in the culture medium [62], [63]. However, the glucose dependency did not apply to phagocytosis of *P. aeruginosa* opsonized with polyclonal rabbit serum, latex particles, unopsonized zymosan, or RBCs opsonized with IgG or IgM and complement. Although we only examined COZ phagocytosis and used only two macrophage cell lines here, our findings suggest that glucose availability may be important for a wider range of actin-dependent processes and monocyte cell types. One rarely discussed process in this context is macropinocytosis activity, which also requires a dynamic actin cytoskeleton. Macropinocytosis activity of macrophages is AMPK-mediated and induced by low glucose conditions [64]. In turn, macropinocytosis may contribute to nutrient uptake capacity, although glucose import probably occurs mainly through GLUT in LPS-stimulated macrophages [6]. Here we would like to speculate that the differences in membrane ruffling (not shown) and recovery of phagocytic activity upon reintroduction of glucose (75% vs. 45%) that we observed between Maf-DKO and RAW 264.7 cells may have to do with the extend of macropinocytosis activity - and metabolic regulation thereof - in these cell lines. It is of note here that phagocytosis and macropinocytosis are mechanistically related processes, and – especially in the initiation phase – also share many morphological features. Alteration of the actin organization, due to loss of MafB, may also explain the differences between the two types of macrophages studied here [65].

Why is presence of glucose so important for formation of actin rich protrusions and spreading and other functional activities of LPS-stimulated macrophages? One possible answer is that there is a direct link to local ATP-production, even though global ATP levels were not affected under the conditions applied. Commonly, when glycolytic ATP-production is disturbed, cells adapt to the use of other substrates such as fatty acids and amino acids (especially glutamine) for ATP production. It has been shown that thioglycollate elicited mouse peritoneal macrophages (which are partially M2-polarized) utilize fatty acids to fuel phagocytosis, especially when glucose is limiting. However, in LPS-stimulated macrophages, the mitochondrial route for ATP-production from fatty acid oxidation is overtly downregulated [8]. This suggests that LPS-stimulated macrophages do not have many alternative sources for fast supply of ATP. Moreover, the intracellular distribution of ATP may play a role. We and others have shown that adenylate kinase or creatine kinase catalyzed phosphotransfer reactions may help in the local ATP supply that is needed for remodeling of the cortical areas of cells during phagocytosis and migration [27], [28], [66], [67]. Since it is generally accepted that actin-rich membrane structures such as filopodia, lamellipodia, ruffles, and maybe also the phagocytic cup, are too thin to contain mitochondria, glucose and its breakdown via glycolysis may be the only source for local ATP production in these structures in LPS-stimulated macrophages. In other cell types, glycolytic enzymes such as aldolase and GAPDH have been shown to be compartmentalized in cortical actin structures, such as pseudopodia, invadopodia, and lamellipodia, and to associate with the actin cytoskeleton [37], [39], [40], [68], [69]. Although very little is known about the proteome of the early forming phagocytic cup, proteomic studies of the phagosome have also identified a role for GAPDH in phagocytosis [70]. A role for glucose catabolism via glycolysis in local - not global - ATP homeostasis may thus explain our findings. A second metabolic role for glucose could be the supply of intermediates in reactions that supply fatty acids and phospholipids for direct local incorporation into the cell membrane [71]. This process is important for maintaining membrane fluidity and for supply of membrane components during formation of protrusions or maturation of the phagocytic cup and movement of the phagosome, respectively. In addition, LPS-stimulated macrophages secrete inflammatory compounds such as IL-1, IL-6, and TNFα. High glycolytic activity may be essential for the synthesis and post-translational modifications of these compounds, as has been shown for quiescent fibroblasts which maintain high glycolytic activity for the synthesis of extracellular matrix proteins [72].

Striking, although considered a gentler way of modulating cell metabolism, since glucose is still available, 2-DG treatment induced cell death earlier than glucose deprivation in our unstimulated cells, although it had a less severe effect on LPS-induced morphodynamics. This raises the possibility that, apart from providing fuel and anabolic material, glucose could affect cellular morphodynamics through another (non-metabolic) mechanism. Indeed, several studies have revealed a direct role for glucose metabolism in posttranslational modification of proteins and signaling to the actin cytoskeleton. Although not known for complement receptor 3, other macrophage receptors that are involved in phagocytosis and adhesion to the extracellular matrix, like CD36, FcγRIII, and CR5a, contain sites for N-linked glycosylation [73]–[75]. 2-DG has been shown to interfere with (i.e. poison) N-linked glycosylation in tumor cells, a process that could be reversed by exogenous addition of mannose [76]. It may, therefore, be possible that 2-DG interfered with normal macrophage receptor expression and function. Michl et al. [42] suggested that 2-DG interferes with reactions that link Fcγ- and C3-receptors with the intracellular contractile apparatus, based on the observation that cellular ATP levels were not disturbed after 2-DG treatment and that the inhibitory effect of 2-DG on phagocytosis was reversed upon addition of mannose or high glucose concentrations. Furthermore, in studies on glucose-induced insulin secretion in pancreatic islet cells and other insulin-secreting cells, such as INS-1, HIT, and MIN6 cells, glucose metabolism has been implicated in the activation of the small GTPase Cdc42, an upstream regulator of actin remodeling. Nevins and Thurmond [77] showed in MIN6 β-cells, that glucose stimulation promotes actin cytoskeletal remodeling by causing alterations in the cycling of Cdc42 between its active GTP-bound and inactive GDP-bound form. Transient activation involves the carboxylmethylation of Cdc42, and occurs rapidly (within 15–30 sec) [78], while deactivation of Cdc42 involves glycosylation of this small RhoGTPase and correlates with transient depolymerization of cortical actin [77]. This suggests that glucose regulates the cortical actin network through modulation of Cdc42 cycling. Transient activation of Cdc42 leads to activation of PAK1 and then Rac1 (another small GTPase involved in actin cytoskeletal remodeling) within 15 minutes after glucose exposure [79]. Finally, Uenishi et al. [80] have recently shown that glucose activates N-WASP via Cdc42 and induces its translocation to the cell membrane of insulin-secreting clonal pancreatic β-cells (MIN6-K8 β-cells). Moreover, glucose stimulation caused LIMK1-mediated phosphorylation and deactivation of cofilin via Cdc42 and PAK1. The timing of these effects may explain why acute removal of glucose from the culture medium had a markedly inhibitory effect on phagocytosis and also why reintroduction of glucose instantaneously restored phagocytosis capacity in our study. Interestingly, LPS signaling to the cytoskeleton also involves the Cdc42-PAK1-Rac1-LIMK1-pathway [81]. LPS stimulation additionally increases glucose uptake and metabolism via PI3K/Akt which are signaling molecules upstream of Cdc42 and Rac1 [82]–[85]. Therefore, by incorporating glucose as signaling molecule, the metabolic changes that are induced by LPS may serve to reinforce the functional changes that macrophage must undergo in order to fulfill their function in host defense and tissue homeostasis.

In summary, our results further establish a pivotal role for glucose and its breakdown via glycolysis in the control of morphodynamic activity in LPS-stimulated macrophages. Based on findings in other cell systems, we consider it likely that for exerting this role, the multitalented properties of glucose as metabolic precursor and molecule for use in post-translational modification of cytoskeletal (associated) structural proteins, receptors or signaling proteins are being used. More in depth investigation is required to further unravel these glucose-related events that control the activities of macrophages.

## Supporting Information

Figure S1 **Oxygen consumption in RAW 264.7 and Maf-DKO macrophages.** Oxygen consumption was measured in suspensions of 1×10^6^ cells on an Oroboros Oxygraph-2k respirometer. RAW 264.7 and Maf-DKO cells were analyzed in parallel on the same day. The basal oxygen consumption was measured where after oligomycin, FCCP, and rotenone was added successively in order to determine the leak respiration, maximal respiration (Max), and residual oxygen consumption (Res). Columns represent means ± SEM of four experiments.
(TIF)

Figure S2 **Macrophages require glucose for phagocytosis of COZ.** RAW 264.7 and Maf-DKO cells were incubated for the indicated times in control medium, or medium containing 2.5 µM oligomycin and 25 mM glucose (A), 10 mM 2-DG and 25 mM glucose (B), or 10 mM galactose and no glucose (C) and stimulated o/n with 100 ng/ml LPS. Phagocytosis efficiency was determined by incubating cells in the respective media with FITC-labeled complement opsonized zymosan (COZ) particles for 30 min and analyzing samples by FACS. Values represent normalized means ± SEM of three independent experiments performed in triplicate. (*p<0.05, **p<0.01, ***p<0.001; one-sample t-test).
(TIF)
