# Supplementary figures and images for: Glucose Controls Morphodynamics of LPS-Stimulated Macrophages
Source: PLoS One. 2014 May 5;9(5):e96786. doi: 10.1371/journal.pone.0096786 (PMC4010488; doi:10.1371/journal.pone.0096786)

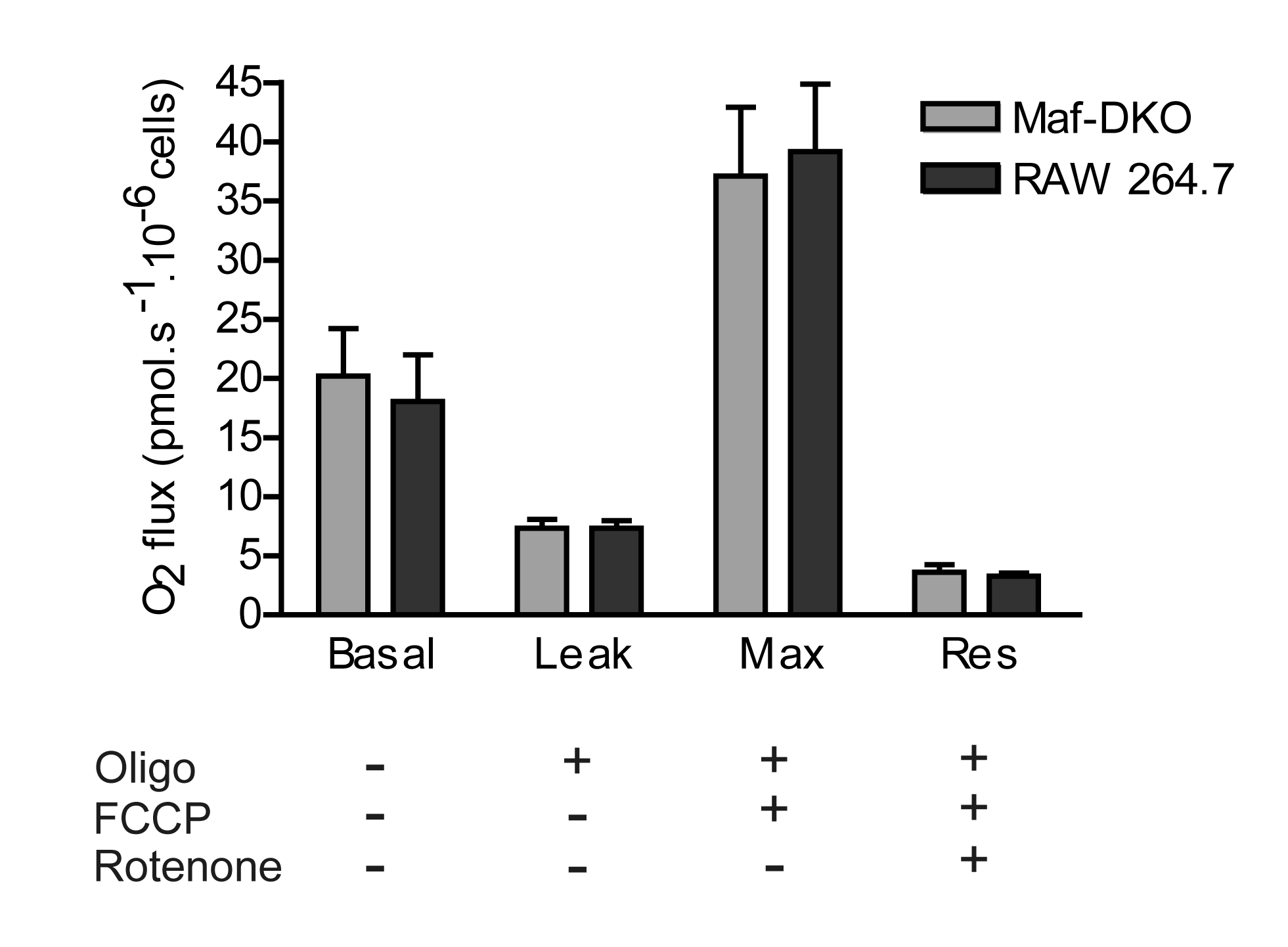

Supplement: Figure S1 — Oxygen consumption in RAW 264.7 and Maf-DKO macrophages. Oxygen consumption was measured in suspensions of 1×10^6^ cells on an Oroboros Oxygraph-2k respirometer. RAW 264.7 and Maf-DKO cells were analyzed in parallel on the same day. The basal oxygen consumption was measured where after oligomycin, FCCP, and rotenone was added successively in order to determine the leak respiration, maximal respiration (Max), and residual oxygen consumption (Res). Columns represent means ± SEM of four experiments. (TIF) [file pone.0096786.s001.tif]

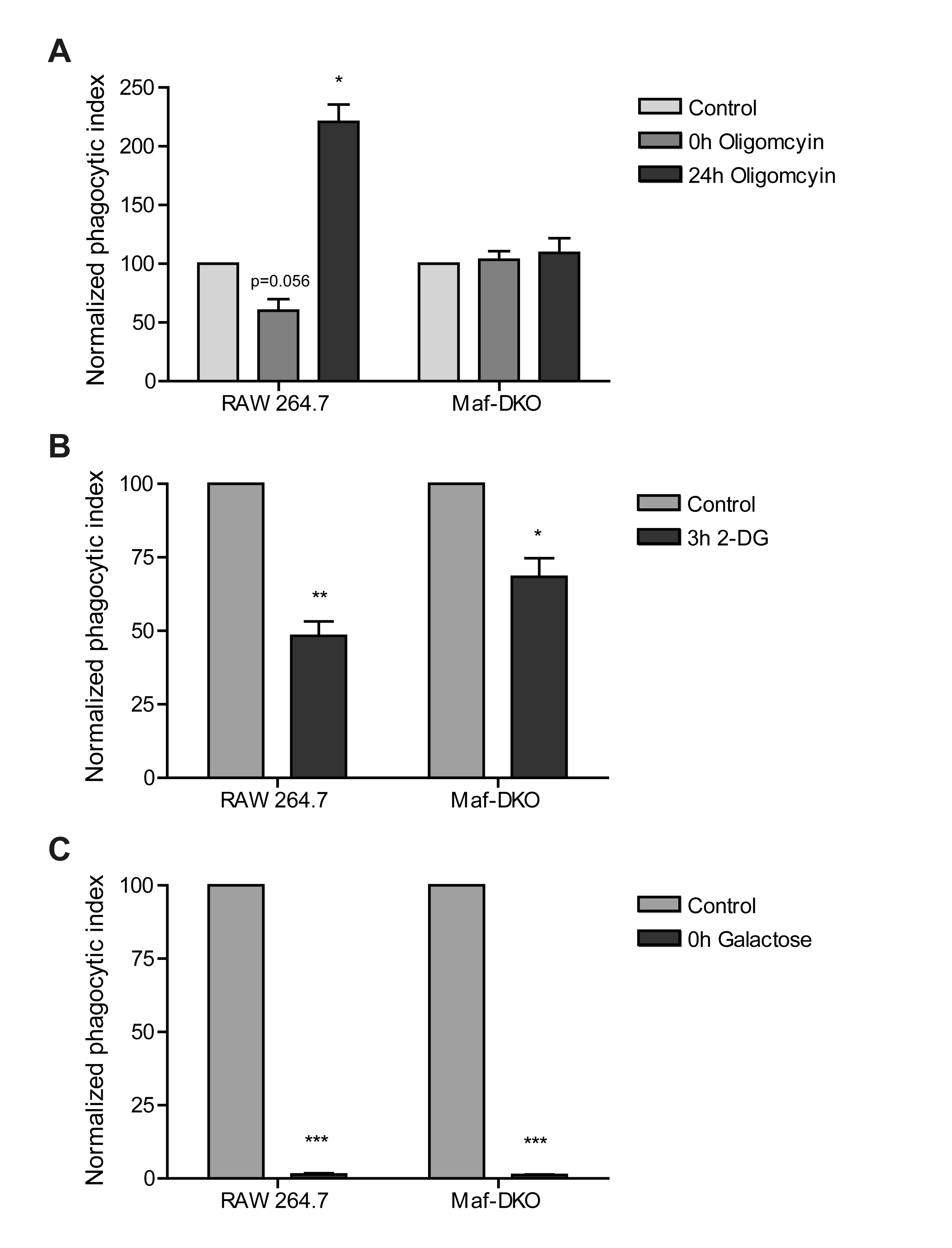

Supplement: Figure S2 — Macrophages require glucose for phagocytosis of COZ. RAW 264.7 and Maf-DKO cells were incubated for the indicated times in control medium, or medium containing 2.5 µM oligomycin and 25 mM glucose (A), 10 mM 2-DG and 25 mM glucose (B), or 10 mM galactose and no glucose (C) and stimulated o/n with 100 ng/ml LPS. Phagocytosis efficiency was determined by incubating cells in the respective media with FITC-labeled complement opsonized zymosan (COZ) particles for 30 min and analyzing samples by FACS. Values represent normalized means ± SEM of three independent experiments performed in triplicate. (*p<0.05, **p<0.01, ***p<0.001; one-sample t-test). (TIF) [file pone.0096786.s002.tif]
